# DACS: from compound collections to rationally designed HTS library

**DOI:** 10.1186/1758-2946-6-S1-P24

**Published:** 2014-03-11

**Authors:** Bernd Rupp, Raed Al-Yamori, Martyna Pawletta, Michael Lisurek, Ronald Kühne

**Affiliations:** 1 Structural Biologie, AG Computational Chemistry/Drug Design, Leibniz-Institut für Molekulare Pharmakologie (FMP), Berlin, D-13125, Germany

## 

DACS (Database of available chemical compounds) was developed to design a HTS collection or focused libraries for the FMP Screening Unit. But the dramatically increase of unique compounds of one order of magnitude from less than 10 to around 30 to 40 million compounds currently and approximately hundred million compounds in the next years requires a reorganization and redevelopment of the storage and searching strategy. To manage such a massive amount of data a relational database for registration and normalization of vendor catalogs was developed. This database contains the highly redundant data of the vendor catalogs and is converted in a second step into the non-redundant Unique Structure Database which represents a data warehouse combining vendor data, structural descriptors and in-house classification tools including our earlier developed ADMET- and reactivity filters as well as our in-house fragment-based fingerprints used for library design tasks [1]. The management of both database systems is part of a new developed Java application, which handles the user management for the data upload in the Registration Database and the conversion into the Unique Structure Database. Moreover a first version of a web service is in preparation. This service allows the scientist not only to search for compounds and fragments in the Unique Structure Database but also to combine such a search with the FMP tools to classify compounds for their usability in biological assays.

## References

[B1] Lisurek M Rupp B Wichard J Neuenschwander M von Kries JP Frank R Rademann J Kühne R Design of chemical libraries with potentially bioactive molecules applying a maximum common substructure concept Mol Divers 2010 14 2 401 408 10.1007/s11030-009-9187-z 19685275 PMC7089384

